# Development of a Non-invasive Methodology for the Assessment of Muscle Fibre Composition

**DOI:** 10.3389/fphys.2019.00174

**Published:** 2019-03-11

**Authors:** Tobias Winkler, Falk Mersmann, Philipp von Roth, Ralf Dietrich, Stefanie Bierbaum, Adamantios Arampatzis

**Affiliations:** ^1^ Center for Musculoskeletal Surgery (CMSC), Charité Universitätsmedizin Berlin, Berlin, Germany; ^2^ Berlin Brandenburg Center for Regenerative Therapies (BCRT), Charité Universitätsmedizin Berlin, Berlin, Germany; ^3^ Department of Training and Movement Sciences, Humboldt-Universität zu Berlin, Berlin, Germany; ^4^ Berlin School of Movement Science, Humboldt-Universität zu Berlin, Berlin, Germany

**Keywords:** fast twitch fibres, muscle power, diagnostic tool, muscle biopsy, soleus muscle

## Abstract

The percentage area of fast twitch fibres of a muscle is a major determinant of muscle mechanical power and, thus, an important biomarker for the evaluation of training processes. However, the invasive character of the assessment (muscle biopsy) limits the wide application of the biomarker in the training praxis. Our purpose was to develop a non-invasive method for the assessment of fast twitch fibre content in human soleus muscle. From a theoretical point of view, the maximum muscle mechanical power depends on the fibre composition, the muscle volume and muscle specific tension. Therefore, we hypothesised that the percentage area of type II fibres would show a correlation with the maximum muscle mechanical power normalised to muscle volume and specific muscle contractile strength (i.e., plantar flexion moment divided by muscle cross-sectional area). In 20 male adults, the percentage area of type II fibres, volume and maximum cross-sectional area of the soleus muscle as well as the maximum plantar flexion moment and the maximum mechanical power were measured using muscle biopsies, magnetic resonance imaging and dynamometry. The maximum mechanical power normalised to muscle volume and specific muscle contractile strength provided a significant relationship (*r* = 0.654, *p* = 0.002) with the percentage area of type II fibres. Although the proposed assessment parameter cannot fully replace histological measurements, the predictive power of 43% can provide a relevant contribution to performance diagnostics in the training praxis.

## Introduction

Athletic training is a dynamic process that triggers specific adaptions within the human musculoskeletal system resulting in an increase in functional performance. An important determinant of athletic performance in several disciplines is the muscle fibre type composition (i.e., slow vs. fast twitch fibres; Saltin et al., 1977; Horowitz et al., 1994; Harber et al., 2002; Kohn et al., 2007). Slow twitch (type I) fibres are characterised by a high content of mitochondria and myoglobin, a high degree of capillarisation, long excitation time and low maximum shortening velocity (Bottinelli et al., 1991, 1994). Fast twitch (type II) fibres are characterised by a high ATPase activity, high content of creatine phosphate and glycogen, short excitation time and high maximum shortening velocity (Bottinelli et al., 1991, 1994). As a consequence, the force-velocity curve of type II fibres shows a less steep decrease with increasing velocity compared to type I fibres and a threefold higher maximum mechanical power (Bottinelli et al., 1991; Gilliver et al., 2009). Specific muscle training that favours the hypertrophy of the type II fibres may be important in activities where high muscle power production (e.g., sprinting or jumping) and high rates of force development (e.g., balance responses after unexpected perturbations) are needed. Resistance exercise can introduce a more pronounced hypertrophy in type II compared to type I fibres (Costill et al., 1979; MacDougall et al., 1980; Aagaard et al., 2001), increasing the percentage area of type II fibres during hypertrophic adaptation without any changes in the numerical fibre distribution within a muscle. In this manner, the percentage area of type II muscle fibres can serve as an important biomarker for the evaluation of training processes. However, this biomarker is practically not available as an accompanying diagnostic measure, since its analysis relies on an invasive procedure (i.e., muscle biopsy). A non-invasive method for the assessment of muscle fibre type composition would be an important diagnostic tool in both athletes and non-athletes.

Several studies have tried to quantify the fibre type distribution, mostly of the knee extensors, according to force-velocity and power-velocity curves (Clarkson et al., 1982; Froese and Houston, 1985; Ryushi and Fukunaga, 1986; Johansson et al., 1987; Larsson and Moss, 1993; Suter et al., 1993; Aagaard and Andersen, 1998). Some of these studies found significant relationships between fibre type distribution and the maximum moment (or torque) or the maximum mechanical power, during isokinetic contractions (Ryushi and Fukunaga, 1986; Johansson et al., 1987; Suter et al., 1993; Aagaard and Andersen, 1998); others could not confirm these relationships (Clarkson et al., 1982; Froese and Houston, 1985; Larsson and Moss, 1993). The divergent results of the above studies can be explained by methodological and conceptual deficits. Muscle fibre length, muscle volume and specific tension (i.e., muscle force normalised to the muscle cross-sectional area) have not been considered in all these studies despite their essential influence on the force-velocity and power-velocity curves. Therefore, significant relationships between fibre type distribution and force-velocity or power-velocity parameters can only be expected in groups of individuals with very homogeneous muscle fibre length, muscle volume and specific tension.

From a theoretical point of view, the maximum mechanical power of a muscle depends on muscle volume, muscle specific tension and the percentage area of type II fibres. In order to establish a causal relationship between the percentage area of type II fibres and the maximum mechanical power, the latter has to be normalised to muscle volume and specific tension. The maximum mechanical muscle power is achieved at approximately 30% of the maximum shortening velocity (Bottinelli et al., 1991; Gilliver et al., 2009) and can be assessed during maximal isokinetic contractions (Iossifidou and Baltzopoulos, 2000), while muscle volume can be determined using imaging techniques. Yet, muscle specific tension can only be estimated *in vivo*, accepting several simplified assumptions and an enormous experimental effort, making it impractical for a wide application. However, specific muscle contractile strength (i.e., joint moment normalised to muscle cross-sectional area) could be a usable surrogate measure in this regard. Our purpose in the current study was to develop a non-invasive method for the assessment of muscle fibre composition in human soleus muscle. We hypothesised that the percentage area of type II fibres would show a high relationship with the maximum muscle mechanical power normalised to muscle volume and specific muscle contractile strength.

## Materials and Methods

### Experimental Protocol

Twenty male sport-active adults (height: 177 ± 6 cm, mass: 74 ± 9 kg, age: 25 ± 8 years) were recruited for the study. In all participants, maximum isometric plantar flexion moment, maximum mechanical power, volume, cross-sectional area and percentage area of type II fibres of the soleus muscle were measured. The specific contractile strength of the soleus muscle was assessed by dividing the maximum plantar flexion moment by the soleus maximum cross-sectional area. All participants gave informed consent to the experimental procedure, which was approved by the Charité Universitätsmedizin (Berlin, Germany) ethics committee according to the rules of the Declaration of Helsinki.

### Muscle Biopsies and Histological Analyses

For fibre distribution analysis, all subjects underwent a fine needle biopsy of the right soleus muscle. All biopsies were harvested under sterile conditions in the operation theatre. Following disinfection and sterile draping, the entry to the soleus muscle was identified using ultrasound lateral at the site of best exposition of the muscle emerging from underneath the gastrocnemius. A stab incision of about 5 mm was performed after local anaesthesia of the skin and subcutaneous tissue with 1% lidocaine and the muscle specimen was taken with a fine needle biopsy device under ultrasound guidance (C.R. BARD Biopsie-System Magnum Core HS, C. R. Bard Inc., Karlsruhe Germany). The muscle specimens were embedded in Tissue-Tek (Sakura Finetek Germany, Staufen, Germany) and transferred into nitrogen-cooled isomethylbutane (Sigma, Taufkirchen, Germany) for 2 min and 5 μm cryosections perpendicular to the fibre axes were produced on a cryotome.

Native cryosections were fixed in cooled acetone for 10 min and washed in phosphate-buffered saline (PBS) twice for 5 min each. Sections were then incubated in 2% horse serum (Vector, Eching, Germany) diluted in PBS for 30 min at room temperature. Subsequently, the primary antibody was applied for 1 h at 37°: anti-myosin fast (1:4,000, #M4276) or anti-myosin slow (1:10,000, #M8421 – Sigma, Taufkirchen, Germany). Following washing twice with PBS for 5 min, the secondary antibody was applied (anti-mouse, rat adsorbed, biotinylated, made in horse, 2% antibody diluted in 2% horse serum in PBS, AK-Standard-Kit AK-5000, Vector, Eching, Germany). Next, sections were washed twice with PBS for 5 min and the avidin-biotin complex was applied for 50 min at room temperature (AP-Standard-Kit AK 5000, Eching, Germany). Finally, sections were washed twice with PBS for 5 min and a nucleus staining (Mayers Haemalaun method) was performed.

**Figure 1:**
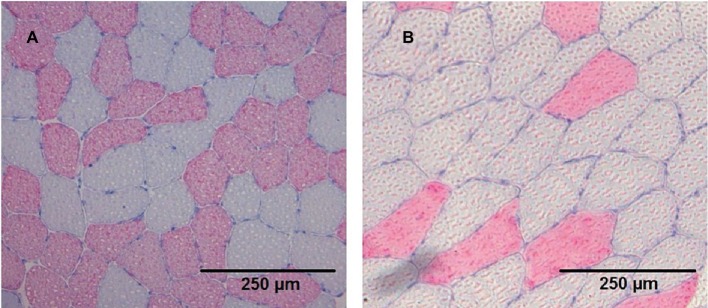
Cryosection stained (anti-MHC II) images from two different participants **(A** and **B)** showing the slow type I (grey) and fast type II (red) fibres.

Images of cryosections were recorded using a Leica DMRB light microscope (Leica, Wetzlar, Germany) equipped with an AxioCam MRc [Carl Zeiss, Göttingen, Germany (Figure 1)]. The absolute area of fast myosin heavy chain (fastMHC) and slow myosin heavy chain (slowMHC) was measured using ImageJ 1.45 s (National Institute of Health, Bethesda, Maryland, USA).

The percentage area of type II muscle fibres was calculated as follows:

$$\mathrm{Area}\%\left[\mathrm{Type}\;II\right]=\frac{\mathrm{Total}\;\mathrm{area}\;\left[\mathrm{Type}\;II\right]}{\mathrm{Total}\;\mathrm{area}\;\left[\mathrm{Type}\;I\right]+\mathrm{Total}\;\mathrm{area}\;\left[\mathrm{Type}\;II\right]}\times 100$$

### Measurement of Muscle Volume and Cross-Sectional Area

Transverse plane magnetic resonance images (MRI) were obtained from the right leg of every participant between the femur condyles and the calcaneal tuberosity (T1 vibe scan, slice thickness 1.8 mm, no inter-slice spacing, echo time 1.18 ms, repetition time 3.11 ms, field of view 244 × 449 mm^2^) lying supine with the knee fully extended in a 1.5 Tesla Magnetom Avanto scanner (Siemens, Erlangen, Germany). To measure the volume of the soleus muscle, the boundaries of the muscles were tracked manually in every image using Osirix (Version 4.0, 64 bit, Pixmeo SARL, Bernex, CH). The muscle volume was calculated from the resulting muscle contours as the integral of the cross-sectional area of the contours along the muscle length, which in turn was measured on the longitudinal axis of the coordinate system (along which the transverse images were obtained) as the distance between the two marginal slices contributing to the muscle reconstruction (Mersmann et al., 2015). From the reconstructed muscle, we determined the maximum cross-sectional area of the soleus muscle.

### Measurement of the Maximum Plantar Flexion Moment and Maximum Mechanical Power

Following a standardised warm-up including 5 min of ergometer cycling, the participants performed three maximal voluntary isometric plantar flexion contractions (MVC) with the right leg at ankle joint angles of 0, 5 and 10° dorsiflexion (0° = tibia perpendicular to the foot) and with a knee joint angle of 140° flexion (0° = knee joint fully extended) on an isokinetic dynamometer (Biodex 3, Biodex Medical Systems, Shirley, NY, USA). This knee joint angle was selected to minimize the contribution of the biarticular gastrocnemii to the resultant ankle joint moment. The highest achieved moment value within the three MVCs was defined as the maximum plantar flexion moment. After the three isometric MVCs, the participants performed three consecutive maximal isokinetic plantar flexion contractions at seven angular velocities of 60, 90, 120, 150, 180, 210 and 240°/s and in an angular range of motion from 20° dorsiflexion to 20° plantar flexion. All contractions (isometric and isokinetic) were performed in randomised order and a 3-min rest was allowed between them.

**Figure 2:**
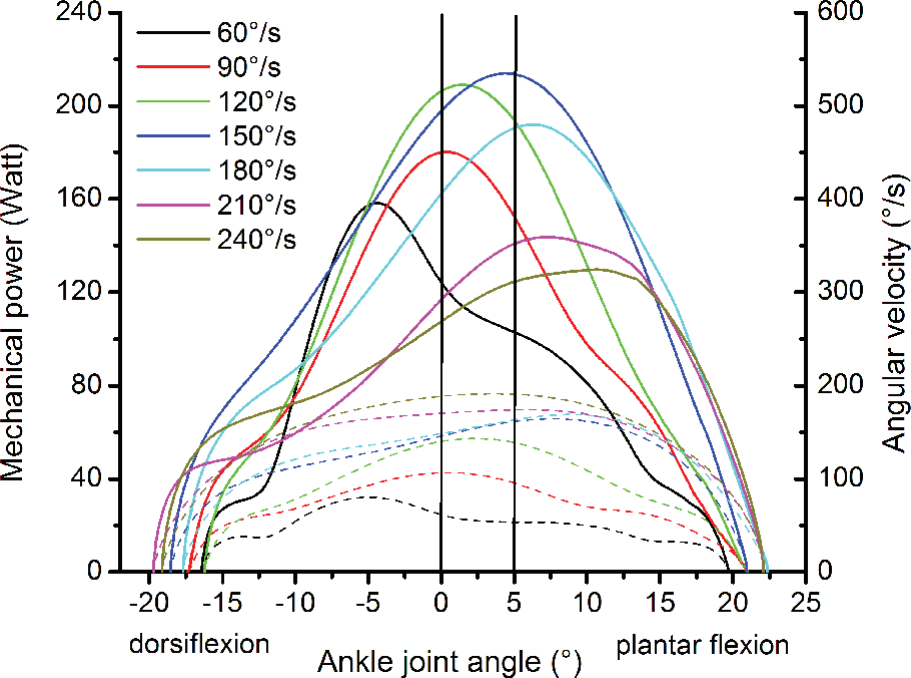
Representative plot of mechanical power (solid lines) and angular velocity of the ankle joint (dashed lines) as a function of the ankle joint angle during the maximum isokinetic contractions of one participant. The two vertical lines show the area where the mechanical power and the angular velocity of the ankle joint were determined.

The resultant moments at the ankle joint were calculated by means of inverse dynamics according to the methodology reported by Arampatzis et al. (2005, 2007) to account for the effect of the misalignment between ankle joint axis and dynamometer axis and the effect of the gravitational forces. The kinematics of the leg and dynamometer footplate were measured using eight cameras (Vicon Motion Systems, Vicon, Oxford, United Kingdom), which recorded 10 reflecting markers (calcaneal tuberosity, lateral and medial malleolus, lateral and medial femoral condyle, greater trochanter, axis of the dynamometer and three additional markers on the footplate) at a frequency of 250 Hz. For the consideration of the gravitational and inertial effects of the foot on the resultant ankle joint moment, we used the data on foot mass and moment of inertia provided by Zatsiorsky and Seluyanov (1983). The effects of the gravitational and inertial forces of the dynamometer footplate on the resultant ankle joint moment were assessed by moving the footplate at the seven investigated angular velocities and at the same angular displacement as during the isokinetic MVCs but without any additional load (Arampatzis et al., 2007). The contribution of the antagonistic moment of the tibialis anterior muscle was considered by establishing a relationship between electromyographic activity and exerted moment of the tibialis anterior while working as agonist (Mademli et al., 2004). The mechanical power of the ankle joint was calculated as the product of the ankle joint moment and ankle joint angular velocity measured by the kinematic analysis of the leg. The highest average mechanical power value between 0 and 5° plantar flexion within the three consecutive contractions was defined as the maximum ankle joint power at each angular velocity. The range between 0 and 5° ankle joint angle was chosen, because in this range the angular velocity of the ankle joint was quite constant in all investigated isokinetic MVCs (Figure 2).

### Statistics

The comparison of the absolute area covered by type I and type II fibres was checked using a paired sample t-test. To investigate the relationship of the percentage area of type II fibres to the maximum mechanical power and to maximum mechanical power normalised to the soleus muscle volume and specific muscle contractile strength, the Pearson correlation coefficient was used. The level of significance for all statistical tests was set to *α* = 0.05.

## Results

**Figure 3:**
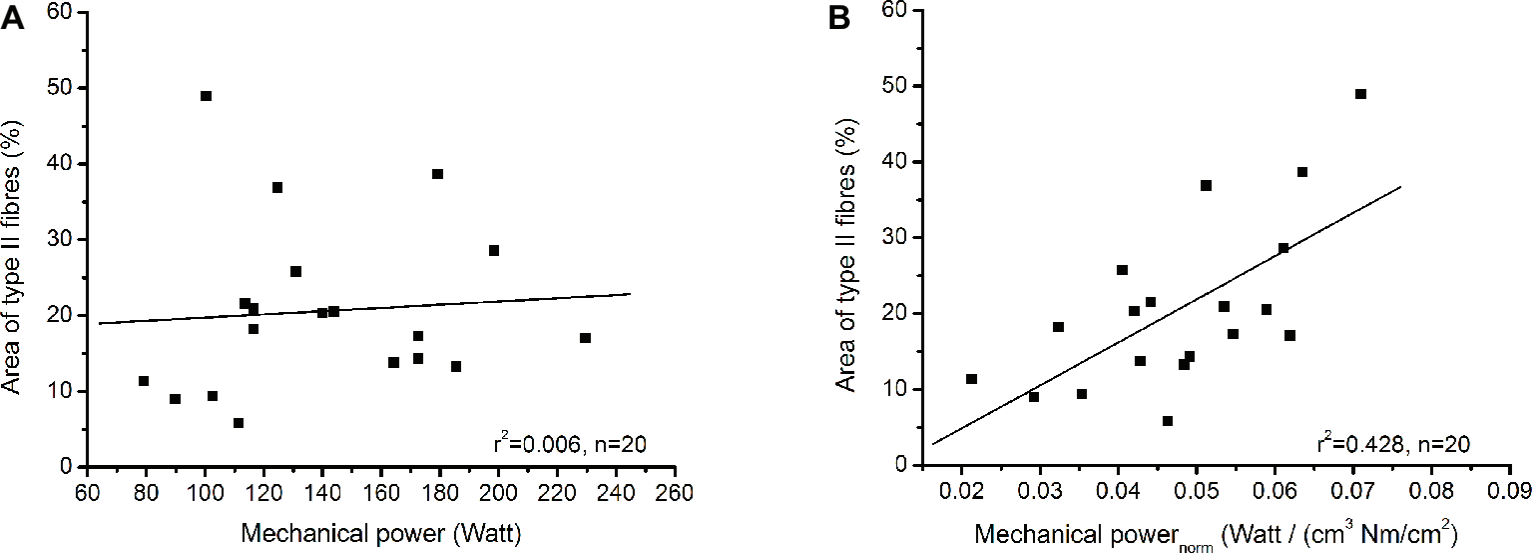
**(A)** Relationship between percentage area of type II fibres and maximum mechanical power. **(B)** Relationship between percentage area of type II fibres and maximum mechanical power normalised to muscle volume and specific contractile strength.

Table 1 shows the examined functional and morphological parameters of the soleus muscle as well as the absolute areas of the biopsy sections covered by type I and type II fibres. The absolute area covered by type I fibres was significantly larger (*p* < 0.001) compared to type II fibres (Table 1) and the average value of the percentage area from fibre II was 20.6 ± 11.1%. Muscle volume of the soleus muscle correlated moderately to maximum mechanical power (*r* = 0.459, *p* = 0.042), but there was no significant correlation between specific contractile strength and maximum mechanical power (*r* = 0.116, *p* = 0.626). The maximum mechanical power did not show any relationship with the percentage area of type II fibres (*r* = 0.078, *p* = 0.754), but the maximum mechanical power normalised to muscle volume and muscle contractile strength provided a significant correlation (*r* = 0.654, *p* = 0.002) with the percentage area of type II fibres (Figure 3).

**Table 1:** Investigated functional and morphological parameters (mean ± standard deviation).

Parameter	
Maximum ankle joint moment (Nm)	189 ± 38
Maximum mechanical power (Watt)	138 ± 40
Angular velocity at maximum mechanical power (°/s)	139 ± 31
Volume of soleus muscle (cm^3^)	483 ± 84
Maximum cross-sectional area of soleus muscle (cm^2^)	30 ± 4
Average absolute area covered by type I fibres (mm^2^)	2.46 ± 1.50^*^
Average absolute area covered type II fibres (mm^2^)	0.59 ± 0.50^*^

* Statistically significant (p < 0.001) differences between type I and type II fibres.

Note that the absolute area refers to the area covered by the respective fibre type in the biopsy section.

## Discussion

The proposed non-invasive assessment parameter (i.e., mechanical power normalised to muscle volume and specific contractile strength), which was based on functional and morphological muscle measures, provided a relevant prediction of the percentage area of fast type II fibres in human soleus muscle. The parameter explains about 43% of the variability in the percentage area of type II fibres. Our theoretical consideration was based on the fact that the maximum mechanical power as a functional muscle parameter depends not only on the fibre composition but also on the muscle volume and muscle specific tension. Muscle volume predicts the muscle mass and the specific tension characterises the contractile quality of the muscle and therefore, both affect the maximum muscle power production. We hypothesised that the maximum mechanical power normalised to muscle volume and specific muscle strength would provide an association with the percentage area of type II fibres, which was confirmed by our results. Although our approach cannot fully replace histological measurements, it can be used in the broad training praxis due to its non-invasive nature.

Strength training is an important part of the training process (Delecluse, 1997; Millet et al., 2002) and can affect the distribution of fibre isoforms, because fast fibres can exhibit a more pronounced hypertrophy than slow fibres in response to specific types of loading (Costill et al., 1979; MacDougall et al., 1980; Aagaard et al., 2001). A non-invasively determined parameter that predicts changes in fibre II distribution would be of great interest within the evaluation of training-induced muscle adaptation, not only in the athletic field but also in rehabilitation programs, due to the crucial contribution of type II fibres to the rate of force development, which is essential for the return to sport (Buckthorpe and Roi, 2018). Muscle biopsies are hardly applicable in the training praxis, because the muscle is injured by taking the biopsy. A biopsy-induced muscle injury would negatively affect the training process, the exercise-related adaptation and performance improvement. The proposed assessment parameter can be determined easier compared to the muscle biopsy method and, more importantly, without any muscle injury. Isometric and isokinetic measurements on a dynamometer are already widely used in strength diagnostics. In our study, the measurement of muscle volume involved the reconstruction of the muscle from MRI scans. A complete muscle reconstruction is still time consuming and cost intensive, which may limit the usability of the method. However, we already provided evidence that the volume of a muscle can be determined with high precision on the basis of the easily determinable maximum anatomical cross-sectional area and the length of the muscle (Albracht et al., 2008; Mersmann et al., 2014, 2015). For the soleus muscle, the precision of this method has been shown to be approximately 5% (Mersmann et al., 2014), indicating an acceptable assessment of muscle volume with easily determined parameters. While using ultrasound for either whole-muscle reconstruction (Weide et al., 2017) or simplified volume prediction (Infantolino and Challis, 2016) may solve the issue of accessibility, the rapid development of deep learning approaches for the automatic segmentation of medical images (Litjens et al., 2017) may soon help to overcome obvious barriers for establishing the assessment in the practical field. While the present study aimed to evaluate the proposed methodology on a single muscle, the analysis of normalised muscle power could provide a valuable estimation of fibre composition in larger muscle groups for the practical field as well. For example, an isokinetic testing of the knee extensors combined with an assessment of quadriceps cross-sectional area and volume would allow for an assessment of overall quadriceps fibre profile, which might be anyway of greater interest to practitioners compared to single muscle fibre composition.

The maximum mechanical power in our experiment was achieved in an average ankle joint angular velocity of 139°/s with a range from 94 to 209°/s within the investigated participants. We used isokinetic contractions between 60 and 240°/s and therefore, we were able to experimentally measure the maximum mechanical power in all participants. The maximum mechanical power within the investigated participants ranged from 79 to 229 W, showing a quite high variability. Similarly, the muscle volume ranged from 329 to 637 cm^3^ and the specific muscle contractile strength from 4.29 to 7.54 Nm/cm^2^, indicating inhomogeneous morphological and muscle specific properties within the participants. This inhomogeneity can explain the only moderate correlation of the muscle volume to the maximum mechanical power and the absence of a significant correlation of the specific contractile strength to maximum mechanical power. The inhomogeneity further explains the absence of a significant correlation between maximum mechanical power and the percentage area of type II fibres in our experiment. From a theoretical point of view, significant relationships between type II fibres distribution and maximum mechanical power can only be expected in groups of individuals with homogeneous features of muscle volume and specific muscle contractile strength.

A limitation of the proposed method is the requirement of a maximum activation of the muscle during the isometric and isokinetic contractions. During maximal voluntary isometric contractions, the muscle activation level is quite high (Arampatzis et al., 2007; Mademli and Arampatzis, 2008), indicating no relevant bias for the assessment of maximal isometric muscle strength. However, during isokinetic contractions, the activation level of the muscle can be decreased (Babault et al., 2002). Although a concentric-induced depression of muscle activation has not been found in all studies (Gandevia et al., 1998), the effect of possibly different activation levels during the isokinetic contractions can affect the maximum mechanical power and remains a limitation of the method. Moreover, in the current study, we investigated the possibility to assess the percentage area of type II fibres of soleus muscle using a combination of non-invasively determined functional and morphological muscle parameters. To evaluate the applicability of the proposed methodology for the detection of training-induced changes of muscle fibre composition was, however, beyond the scope of this study and remains a future challenge.

In conclusion, we found a significant relationship between the percentage area of type II fibres of the soleus muscle and the maximum mechanical power normalised to muscle volume and specific muscle contractile strength. With 43% of the variance in type II fibre area explained, normalised maximum power could perspectively be a useful diagnostic parameter for the training practice, especially in light of recent technological and computational advances simplifying the assessment of muscle volume. However, it cannot completely replace histological measurements.

## Author Contributions

TW carried out and analysed the biopsies and drafted the manuscript. FM participated in data analysis and in drafting of the manuscript. PR carried out and analysed the biopsies. RD and SB carried out the experiments and analysed the data. AA conceived and coordinated the study, participated in data analysis and drafted the manuscript. All authors gave final approval for publication.

### Conflict of Interest Statement

The authors declare that the research was conducted in the absence of any commercial or financial relationships that could be construed as a potential conflict of interest.
